# Histone deacetylase 2 inhibitor valproic acid attenuates bisphenol A-induced liver pathology in male mice

**DOI:** 10.1038/s41598-022-12937-4

**Published:** 2022-06-17

**Authors:** Mohamed A. Al-Griw, Zaynab Osama Alshibani, Rabia Alghazeer, Mohamed Elhensheri, Refaat. M. Tabagh, Areej A. Eskandrani, Wafa S. Alansari, Mahmoud M. Habibulla, Ghalia Shamlan

**Affiliations:** 1 Department of Histology and Genetics, Faculty of Medicine, University of Tripoli, Tripoli, Libya; 2 Department of Zoology, Faculty of Sciences, University of Tripoli, Tripoli, Libya; 3 Department of Chemistry, Faculty of Sciences, University of Tripoli, P.O. Box: 13203, Tripoli, Libya; 4 Department of Research of Oral and Maxillofacial Surgery, Facial Plastic Surgery, University Medical Centre Rostock, 11 Rostock, Germany; 5 Chemistry Department, Faculty of Science, Taibah University, Medina, Saudi Arabia; 6 Biochemistry Department, Faculty of Science, University of Jeddah, Jeddah, Saudi Arabia; 7 Department of Medical Laboratory Technology, Faculty of Applied Medical Sciences, Jazan University, Jazan, Saudi Arabia; 8 Department of Food Science and Nutrition, College of Food and Agriculture Sciences, King Saud University, Riyadh, 11362 Saudi Arabia

**Keywords:** Biochemistry, Cell biology

## Abstract

Accumulating evidence indicates the role of endocrine disruptor bisphenol A (BPA) in many pathological conditions. Histone deacetylase (HDAC) inhibition has potential for the treatment of many diseases/abnormalities. Using a mouse BPA exposure model, this study investigated the hepatoprotective effects of the Food and Drug Administration–approved HDAC2 inhibitor valproic acid (VPA) against BPA-induced liver pathology. We randomly divided 30 adult male Swiss albino mice (8 weeks old; *N* = 6) into five groups: group 1, no treatment (sham control (SC)); group 2, only oral sterile corn oil (vehicle control (VC)); group 3, 4 mg/kg/day of oral BPA (single dose (BPA group)); group 4, 0.4% oral VPA (VPA group); and group 5, oral BPA + VPA (BPA + VPA group). At the age of 10 weeks, the mice were euthanized for biochemical and histological examinations. BPA promoted a significant decrease in the body weight (BW), an increase in the liver weight, and a significant increase in the levels of liver damage markers aspartate aminotransferase and alanine aminotransferase in the BPA group compared to SC, as well as pathological changes in liver tissue. We also found an increase in the rate of apoptosis among hepatocytes. In addition, BPA significantly increased the levels of oxidative stress indices, malondialdehyde, and protein carbonylation but decreased the levels of reduced glutathione (GSH) in the BPA group compared to SC. In contrast, treatment with the HDAC2 inhibitor VPA significantly attenuated liver pathology, oxidative stress, and apoptosis and also enhanced GSH levels in VPA group and BPA + VPA group. The HDAC2 inhibitor VPA protects mice against BPA-induced liver pathology, likely by inhibiting oxidative stress and enhancing the levels of antioxidant-reduced GSH.

## Introduction

Environmental toxicant exposure is a major health concern. Environmental toxicants affect many physiological processes and cause extensive tissues/organs damage^1,2^. Environmental toxicant exposures during intrauterine life, postnatal life, and early life play an important role in determining mature phenotypes and vulnerability to anomalies/morbidities in adult life^3–5^. An endocrine disruptor chemical, bisphenol A (BPA), is commonly used in the production of epoxy resins and polycarbonate plastics^6,7^. Because of its widespread use in the manufacturing of plastic containers for food or refreshments and coatings of nourishment cans, there is an unavoidable risk of BPA exposure^8,9^. Due to this BPA can be detected in the environment, food, and even in human body fluids^10^. BPA concentrations as high as 0.2–106 ng/g in food , up to 2–208 ng/m^3^ in air, and 54–79 μg/cm^2^ in thermal paper are reported^10^. BPA levels can reach 0.5–10.0 μg/L in blood and 59.72 and 4.76 μg/L in urine and placenta, respectively^10^. Ubiquitous BPA exposure and its toxic potential have raised concerns with regard to its adverse effects on different biological systems^11,12^. Increasing evidence indicates that BPA toxicity is tissues/organ specific, which may lead to diseases/abnormalities of a particular organ^13,14^. Thus, due to its ubiquitous exposure and negative health effects, the BPA has attracted greater attention in the field of public health. BPA shows potential acute and sub-chronic toxicity with even short periods of exposure^15,16^. BPA at a dose of 50 mg/kg body weight is reported to affect kidney, liver, and BW at^15^. Similar observations have been made using different BPA doses^15,17^.

Environmental toxicants interrupt normal liver functioning, leading to several diseases/abnormalities^18,19^. Due to its ubiquitous presence, BPA can damage tissues/organs in humans as well as animals^20,21^. BPA may induce apoptosis in mammalian germ cells, osteoblasts and osteoclasts^22–25^.

Reactive oxygen species (ROS) trigger oxidative stress, which is called a genotoxic^26,27^. The generation of ROS, including hydroxyl radicals (HO^−^) and superoxide ion (O_2_^−^), alters enzyme activity, decreases DNA repair, impairs oxygen utilization, and depletes glutathione (GSH). Oxidative damage in responding to environmental toxicant exposure has been reported in many models^4,23,28^. It is a possible mechanism underlying the relationship between BPA exposure and adverse health outcomes^4,8^. BPA exposure causes kidney, brain, and other organ injury by generating oxygen free radicals^29,30^. Evidence is suggestive of generation of reactive oxygen species as a predominant mechanism underlying the association of BPA toxicity with hepatic dysfunction^31^.

Antioxidants play a major role in disease prevention due to their ROS scavenging activity^26^. Reactive metabolite and ROS generation can affect GSH homeostasis^32^. GSH is a significant cell-ensuring biomolecule against synthetic actuated cytotoxicity by immediate or glutathione-S-transferase conjugation with electrophilic compounds^33^. In addition, GSH is a major antioxidant agent, which can perform the immediate or enzymatic (glutathione peroxidase) formation of hydrogen peroxide and lipid hydroperoxides with ROS^34^. The activity of GSH in a substance’s harmfulness is to decide the outcomes of decreasing or exhausting cellular GSH. The ROS in the liver are scavenged by its antioxidant enzymes including, catalase (CAT), superoxide dismutase (SOD) and reduced GSH^29,30^. The liver is a major target of BPA-induced organ injury; liver injury seen in rats exposed to BPA has been shown to be associated with an imbalance in redox status^35^.

Epigenetics play a significant role in health and disease. The state of the histone acetylation/deacetylation balance affects epigenetic alteration, which controls gene expression and affects other cellular properties such as cell migration, survival, regulation, and proliferation^36^. Histone acetylation is under the regulation of HDACs and histone acetyltransferases (HATs)^36^. HATs unwind the chromatin structure by acetylating lysine residues^36^. They enhance transcription as they upregulate the transcription factor binding to nucleosomal DNA; however, they remove acetyl groups from histones, having the opposite effect on chromatin, which means that HDACs can be used as therapeutic targets for reversing aberrant acetylation during cellular events^36^.

The HDACs are inhibited by HDAC inhibitors leading to post-translational acetylation of lysine residues of proteins in nucleus and cytoplasm, altering the activity and functions of these proteins^37,38^. HDAC inhibition has a deep impact on the acetylation of histone proteins, augmenting the expression of genes known to protect against insults^37,38^. Moreover, attenuation of deacetylation also aids acetylation of nonhistone proteins, including signal transduction mediators and transcription factors, thereby regulating localization, interaction and stability of these proteins^39,40^.

BPA exposure results in epigenetic abnormalities^41^. Higher BPA concentrations cause chromatin compaction and impair oxidative stress-induced DNA repair due to inhibition of key repair enzymes^8^. BPA exposure affecting chromatin methylation has also been reported earlier^42,43^. These findings indicate a cytotoxic and genotoxic nature of BPA and may be related to ROS production^8^. In addition, BPA alters several functions such as recognition of DNA damage and its repair, especially that of free radical-induced injury, which occurs via modulation of the chromatin architecture or of the response and repair proteins^8^.

HDAC inhibition shows positive therapeutic potential in many disease models, including inflammatory and autoimmune diseases as well as many neurodegenerative conditions^38,44–47^. The Food and Drug Administration (FDA)-approved HDAC2 inhibitor 2-propyl-pentanoic acid, also known as valproic acid (VPA) is used in both preclinical and clinical settings^48^. Regarding its therapeutic mechanism, VPA inhibits gamma aminobutyric acid inactivation. It has been shown that VPA can inhibit HDACs^49,50^. VPA selectively modulates the activity of HDAC2^51^. The role of HDACis in protecting against oxidative liver damage is not well understood. Although the mechanism underlying liver toxicity is unclear, it is hypothesized that ROS overproduction compromises antioxidant capacity and leads to oxidative stress, which is major risk factor of liver toxicity^52^.

The liver play a crucial role in the detoxification of xenobiotics, both in humans and animals, and therefore, injury to the liver is associated with many disorders/abnormalities^53^. Here we demonstrated that whether BPA exposure disrupts liver function and architecture, oxidative stress, and antioxidative status in male mice. It also determined the therapeutic potential of ROS as a target, as demonstrated here through the use of HDACis.

## Results

### BPA and VPA effects on animal survival

The survival/mortality rates of the experimental groups were compared. No mortality and signs of acute toxicity were noted in all the studied groups. There was no significant difference between the SC and BPA groups.

**Figure 1:**
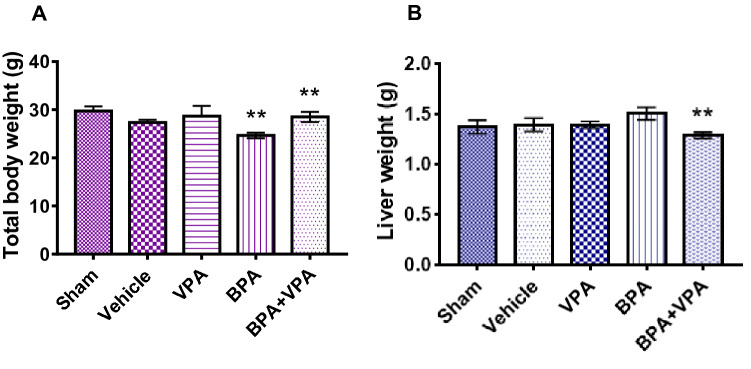
VPA preserved body and liver weights in BPA-exposed mice. (**A**) Quantification of the TBW. (**B**) Quantification of the liver weight. Data are represented as the mean ± SEM (*n* = 6 per group). **P < 0.01. *VPA* valproic acid, *BPA* bisphenol A, *TBW* total body weight, *SEM* standard error of the mean.

HDAC2 inhibitor VPA positively affect the body and liver weights in mice after BPA exposure. To determine the effects of BPA exposure on the total body weight (TBW) and liver weight, the TBW of all experimental groups were monitored throughout the study period and the final body and liver weights recorded at the time of necropsy (Fig. 1). For the TBW, statistical analysis showed a significant difference in the mean TBW between BPA and control (SC and VC) groups (*P* < 0.0014; Fig. 1A), but VC showed no effect compared to SC. For BPA + VPA group, we observed a positive effect on the mean TBW compared to BPA group (*P* < 0.0089; Fig. 1A). The mean liver weight of BPA group increased compared to SC without statistical significance (*P* < 0.16; Fig. 1B). In contrast, VPA treatment after BPA exposure was effective against BPA-induced liver weight reduction (*P* < 0.0096; Fig. 1B) and restored the weight nearly to its normal level.

### HDAC2 inhibitor VPA attenuated liver damage markers in the sera of mice after BPA exposure

**Figure 2:**
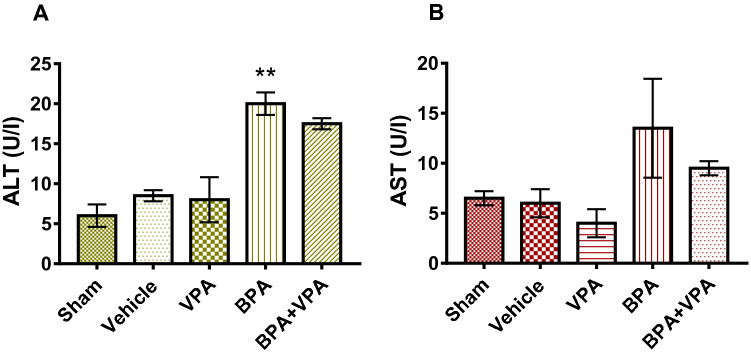
VPA decreased liver damage markers in the sera of BPA-exposed mice. (**A**) Measurement of ALT levels (U/I). (**B**) Measurement of AST levels (U/I). Data are represented as the mean ± SEM (*n* = 6 per group). **P < 0.01. *VPA* valproic acid, *BPA* bisphenol A, *ALT* alanine aminotransferase, *AST* aspartate aminotransferase, *SEM* standard error of the mean.

We investigated the effects of VPA on liver function in mice after BPA exposure by measuring the serum ALT and AST levels, which are markers of liver damage (Fig. 2). Significant ALT increase was noted in the BPA group compared to SC (P < 0.0018; Fig. 2A).

In contrast, we did not detect significant differences in AST levels between groups SC, VC and BPA group (P < 0.153; Fig. 2B). These results showed that BPA has toxic effects on liver function (hepatotoxicity). Compared to the BPA group, the BPA + VPA group showed a decrease in serum ALT and AST levels albeit without statistical significance (Fig. 2A,B).

**Figure 3:**
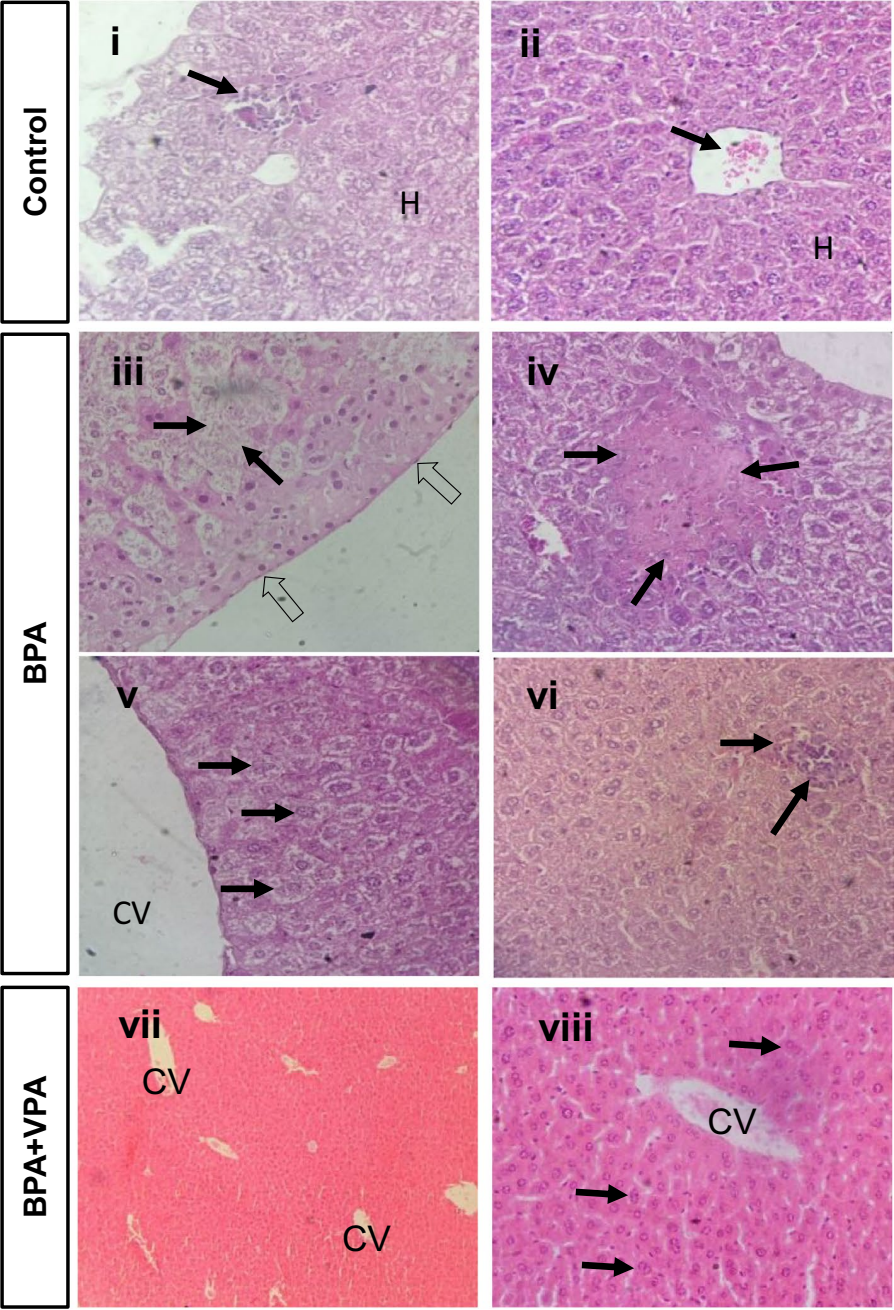
Representative photomicrographs of H&E-stained liver tissue of SC (**i**,**ii**), 3 (**iii**–**vi**), and 5 (**vii**,**viii**). Histopathological features of liver tissue of SC associated with the following outcomes: (**i**) lymphohistiocytic foci (arrow) (lobular focal minimal) with (**ii**) minimal multifocal hyperemia (arrow) and minimal multifocal erythrocyte extravasation. Histopathological features of liver tissue of BPA group associated with the following outcomes: (**iii**) hepatocellular hydropic degeneration (arrows), panlobular, close to Glisson’s capsule (open arrows); (**iv**) hepatocellular coagulative necrosis with cytoplasmic membrane disintegration, cytoplasmic eosinophilic changes, karyopyknosis, and karyolysis, accompanied by lymphohistiocytic infiltration with a few neutrophils (arrows); (**v**) microvesicular, centrilobular hepatocellular steatosis; and (**vi**) lymphohistiocytic infiltration, panlobular multifocal (arrows). Histopathological features of liver tissue of BPA + VPA group associated with the following outcomes: **(vii)** preserved liver architecture, including portal and central veins, predominated by a regular histomorphological, lobular architecture and (**viii**) hepatocyte proliferation and repopulation characterized by irregular and thinking of hepatocyte’s cords and enlargement of hepatocytes, including binucleated and multinucleated hepatocytes (arrows), in addition to multifocal hyperemia and minimal multifocal erythrocyte extravasation. Scale bars = 100 µm; 100 × m H&E staining. *H* hepatocytes, *CV* central vein, *H&E* hematoxylin and eosin.

The HDAC2 inhibitor VPA alleviated liver pathology in mice after BPA exposure. Liver tissue from the SC group showed multifocal, lobular, lymphohistiocytic foci with minimal multifocal hyperemia and minimal multifocal erythrocyte extravasation (Fig. 3, panels i–iv). Compared to controls (sham and vehicle), liver tissue of the BPA group showed prominent hepatocellular degeneration (hydropic, panlobular, multifocal, mild), characterized by increased cytoplasmic granularity, cell swelling, and a pale cell cytoplasm with variable nuclei, usually not accompanied by infiltration, diagnosed as hepatocellular hydropic degeneration. We also found hepatocellular coagulative necrosis (panlobular, multifocal, mild), interpreted as cytoplasmic membrane disintegration, including cytoplasmic eosinophilic changes, karyopyknosis, karyolysis, and complete dissolution of nuclear chromatins of hepatocytes, accompanied by lympho- histiocytic infiltration with a few neutrophils (multifocal, mild). In addition, there was hepatocyte degeneration (centrilobular, multifocal, mild), described as swollen hepatocytes with a pale, eosinophilic, finely vacuolated cytoplasm, interpreted as microvesicular hepatocellular steatosis. In addition, multifocal, lobular, lymphohistiocytic foci with minimal multifocal hyperemia and minimal multifocal erythrocytes extravasation were also seen, concentrated in portal tracts, which were considerably expended (Fig. 3, panels iii–vi). In contrast, liver tissue from BPA + VPA group showed prominent hepatocyte regeneration and repopulation, demonstrated by various morphological changes, predominated by a regular histomorphological, lobular architecture, and hepatocyte proliferation, characterized by irregular, and thinking of hepatocyte cords and hepatocyte enlargement, containing two or more nuclei with prominent nucleoli, defined as binucleated, and multinucleated hepatocytes. Minimal multifocal hyperemia and minimal multifocal erythrocyte extravasation were also seen. Interestingly, histopathological investigation showed no appearance of pathologic hepatocellular necrosis, hepatitis, and/or distinct fibrosis (Fig. 3, panels vii, viii).

### HDAC2 inhibitor VPA decreases hepatocellular apoptosis in mice after BPA exposure

**Figure 4:**
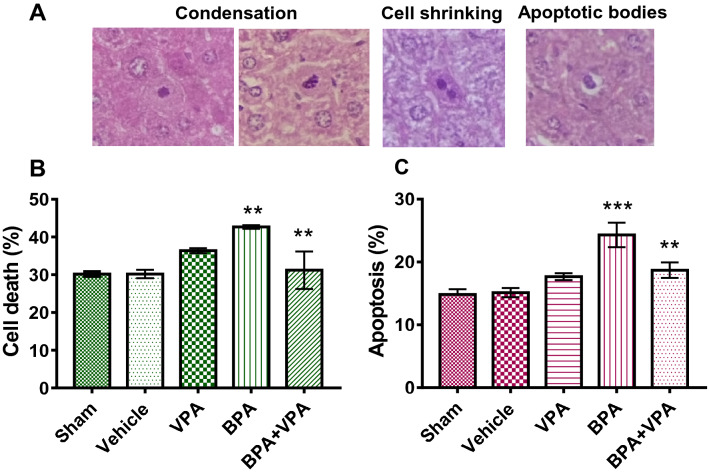
VPA minimizes hepatocellular apoptosis in BPA-exposed mice. (**A**) Representative photomicrographs of H&E-stained liver tissue sections. Morphological microscopic patterns of delayed nuclear condensation, fragmentation, and apoptotic bodies, which were used to determine the cell death profile in each experimental group. Scale bar = 50 µm; 63 × magnification. (**B**) Quantification of hepatocyte death. (**C**) Quantification of apoptotic hepatocytes with fragmented DNA. Data are represented as the mean ± SEM (*n* = 6 per group). **P < 0.01 and ***P < 0.001. *VPA* valproic acid, *BPA* bisphenol A, *H&E* hematoxylin and eosin, *SEM* standard error of the mean.

Apoptosis analysis showed that BPA group showed a significant elevation in the hepatocyte death rate compared to SC (*P* < 0.0012; Fig. 4B); VC did not show any effect under control conditions (data not shown). In contrast, the BPA + VPA group showed a significantly decreased hepatocyte death rate (*P* < 0.0022; Fig. 4B). Liver tissue sections from all experimental groups were stained with H&E (Fig. 4A).

The percentage of apoptosis of hepatocytes was determined using strict morphological criteria^54^. Cytomorphological evidence of the BPA group showed significant hepatocyte apoptosis, represented by prominent chromatin condensation, DNA fragmentation, cytoplasmic vacuolation, and apoptotic body formation, accompanied by cell-related degenerative changes (Fig. 3). SC did not show hepatocyte apoptosis features. Statistically, the BPA group showed a significant augmentation in hepatocyte apoptosis rate compared to SC (*P* < 0.0001; Fig. 4C). the BPA + VPA group showed significantly decreased hepatocyte apoptosis (*P* < 0.0021; Fig. 4C). These results indicated that VPA treatment induces a protective (antidote) effect against BPA-induced cell injury, including hepatocyte apoptosis.

### HDAC2 inhibitor VPA modulates oxidative and antioxidative status in mice after BPA exposure

**Figure 5:**
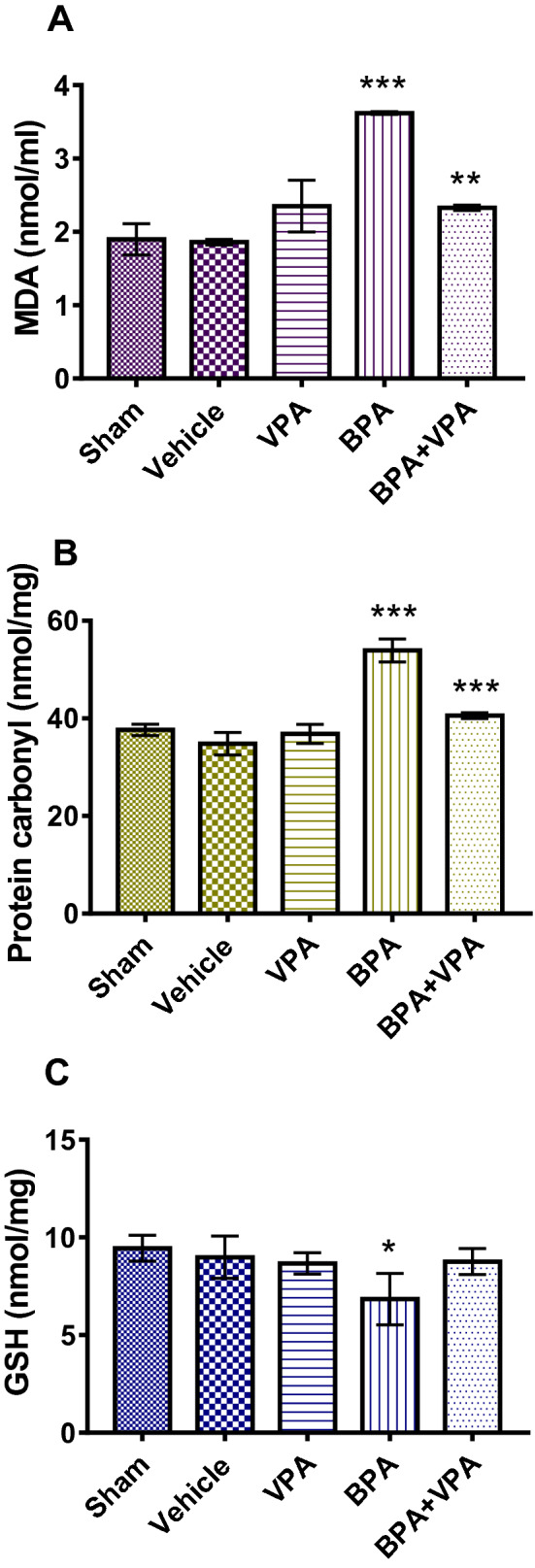
VPA decreased oxidative stress markers MDA and PC and enhanced the antioxidant marker GSH in BPA-exposed mice. (**A**) Measurement of MDA levels (nmol/mL). (**B**) Measurement of PC content (nmol/mg). (**C**) Measurement of reduced GSH (nmol/mg). Data are represented as the mean ± SEM (*n* = 6 per group). *P < 0.05, **P < 0.01, and ***P < 0.001. *VPA* valproic acid, *BPA* bisphenol A, *MDA* malondialdehyde, *PC* protein carbonyl, *GSH* glutathione, *SEM* standard error of the mean.

The BPA group showed significantly increased lipid peroxidation, as revealed by increased MDA levels in the liver tissue, compared to SC (*P* < 0.0001; Fig. 5A). In addition, there was a significant elevation in protein peroxidation, as determined by protein carbonyl (PC) levels (*P* < 0.00001; Fig. 5B). In contrast, PC and MDA levels in the liver tissue of the BPA + VPA group significantly decreased compared to the BPA group and were restored nearly to their normal levels (*P* < 0.0017 and *P* < 0.0006, respectively; Fig. 5A,B).

Impact assessment of VPA’s effect on the antioxidative status in mice after the BPA exposure compared the liver GSH levels in SC. BPA group had lower liver GSH levels matched to SC (*P* < 0.05; Fig. 5C), indicating that VPA treatment enhances GSH levels compared to BPA-untreated mice without statistical significance (*P* > 0.05; Fig. 5C).

## Discussion

This study showed that BPA significantly induces liver pathology, as demonstrated by biochemical tests and histopathological findings. BPA in particular decreases the BW and increases the liver weight compared to controls, parallel to increased levels of liver enzyme indices (AST and ALT levels), hepatocellular apoptosis, hepatocyte injury scores, and oxidative stress indices (MDA and PC levels). There is also a decrease in GSH levels. Many of these noxious effects are attenuated by VPA treatment, which suggests an important role for the HDAC2 inhibitor VPA in BPA-induced injury and also indicates that decreasing oxidative stress and increasing GSH levels could contribute to the protection provided by VPA.

HDAC inhibitors have emerged as potent therapeutic agents in a range of diseases/abnormalities, since they have the ability to accelerate histone acetylation, alter the transcription of associated genes, and protect tissues^36^. Brain cultures of mice exposed to 50 mg/kg of BPA in their diet showed that in the promoter region of Kcc2 (the ion channel responsible for excreting chloride ions to the extracellular fluid), BPA-induced DNA hypermethylation and decreased histone acetylation. In addition, the MeCP2 expression and the ability of BPA to enhance the binding of MeCP2 to the Kcc2 promoter increased, decreasing the expression of Kcc2^55^, indicating a relationship between a decrease in acetylated histone and HAT inhibition and/or HDAC activation after BPA insult, which can be prevented by HDACi treatment.

The HDAC2 inhibitor VPA is widely used for neurological abnormalities such as epilepsy, bipolar disease, and some painful neuropathies^48^. It inhibits HDAC classes I and II, but not class III^48^. Because of their effect on acetylation sites, HDACi could be used as therapeutic agents. Currently, 18 different HDACs have been identified in mammals^36,46^. However, the roles of the different HDAC isoforms and HDACi in models of toxicant-induced liver injury are unclear. Therefore, this study focused on the efficacy of the HDAC2 inhibitor VPA against BPA-induced liver pathology.

Increased serum transaminases are a sign of liver damage and were reported in rats treated with 5 mg/kg/day of BPA, but not with < 5 mg/kg/day of BPA^56^. In male rats treated with ≥ 200 mg/kg/day of BPA, Yamasaki et al. found increased AST activity and increased ALP and glutamyl transpeptidase activities in those treated with 600 mg/kg/day of BPA^57,58^. In this study, 4 mg/kg of BPA significantly raised serum levels of liver enzymes (high levels of ALP and ALT) compared to controls, which are attributed to liver injury. Treatment with VPA after BPA exposure modulated serum AST and ALT levels.

Increasing evidence shows that BPA may be hepatotoxic. BPA exposure is linked to an increased risk of being overweight^59^. Prenatal and early postnatal BPA exposure is correlated with increased BW^9,60–62^. Significant decline in BW was found in the group treated with 0.1 mg/kg of BPA^63^ and ≥ 466 mg/kg/day of BPA. A dose-dependent decrease in relative (> 10%) and absolute (> 22%) liver weights was found at ≥ 466 mg/kg/day of BPA compared to controls^57,58^. In this study, the VPA group was not statistically different from sham and therefore implies a return to baseline, but that significance between the treatment groups was not seen.

The liver plays several roles in detoxification and protects the body from potentially toxic chemicals throughout xenobiotic biodegradation and metabolism. Environmental toxicants disrupt normal liver functioning, causing various diseases^18,19^. As a ubiquitous environmental toxicant, BPA damages tissues/organs in humans and animals^20,21^. BPA is shown to induce apoptosis in different mammalian cells, including, osteoclasts germ cells and osteoblasts^23,25^. HDAC treatment prevented cell loss in many in vitro and in vivo disease models^64–66^. In this study, BPA significantly increased histopathological scores of hepatocytes, they are representative of liver injury, and treatment with VPA after BPA exposure ameliorated liver function, attenuated liver pathology, and decreased hepatocyte apoptosis due to BPA’s noxious effect.

BPA injures the liver primarily by increasing oxidative stress, increasing the inflammatory response, and inhibiting mitochondrial function^63,67^. In mammals, ROS produced as a result of aerobic metabolism exert adverse effects on host cells^68^. These ROS affects the body’s capacity to effectively negate free radicals^69^. Among the body’s many defense mechanisms, oxidative stress is very important, which protects mammalian cells from environmental toxicants^70^. Clinical and experimental studies have reported that the oxidative stress induced by ROS play an important role in liver injury^56,71^. Because of limited information about BPA’s effects on the liver, this study investigated possible hepatotoxic effects of BPA by inducing oxidative stress. We found that BPA alters the body’s oxidative status, characterized by elevated MDA and PC levels. A previous study demonstrated increased liver MDA levels in BPA exposed rats^56^. Other reports also showed increased MDA levels in the testes, brain and kidneys of BPA exposed rats^35,56^. In addition, VPA prevents the peroxidation of membrane lipids and protein carbonylation after BPA-induced liver pathology in rats and is also effective in attenuating acute lung injury caused due to ischemia–reperfusion^72^.

Cellular damage due to interaction between macromolecules such as lipids, proteins, and DNA and ROS can be inhibited by antioxidants. Therefore, overproduction of or unbalanced ROS leads to clinical disorders, which can occur as a disturbance of the redox reactions in cells as an effect of BPA, which, in turn, increases oxidative stress^73^.

GSH is an antioxidant which participates in catalysis of other antioxidants including GSH peroxidase and GSH reductase. A decrease in GSH derails antioxidant defense against free radicals^74^. In addition, decreased GSH levels and increased MDA levels are signs of overproduction of free radicals, which causes lipid peroxidation in the liver^75^.

In this study, we found that the antioxidant capacity in BPA-treated mice was blunted, possibly due to decreased GSH levels. Similarly, a prior study showed a decrease in GSH levels in the livers of BPA-treated rats^56^. Consistent with our findings, studies have also reported liver damage in BPA-treated rats due to decrease in GSH^63,76^. BPA is well-known to generate ROS, which cause damage to many organs^26,30^. Our results, therefore, demonstrated that BPA causes oxidative damage to the liver by derailing the balance between ROS and the antioxidants in the liver and that the HDAC2 inhibitor VPA increases cellular GSH levels in the liver decreased by BPA exposure.

## Conclusion

This work highlights that BPA exposure has detrimental effects on liver tissue architecture of male mice. In addition, our findings also showed that BPA significantly promoted liver oxidative injury by disturbing oxidant/antioxidant status, and ultimately led to liver abnormality as revealed by biochemical test and histological alterations. These findings shed light to realize possible underlying mechanism(s) on the development of a range of abnormalities induced by BPA. Importantly, the concurrent treatment by HDAC2 inhibitor VPA had protective effects on the liver tissue structure, and afforded protection against BPA toxicity. However, we were incapable of finding the precise mechanism by which VPA exerts its actions. Nevertheless, our findings imply that some impacts may be mediated by suppression of oxidative stress markers (MDA and PC) and enhancement of GSH levels upon BPA-mediated liver toxicity. Anyway, our findings suggest that VPA provides effective treatments for BPA-induced liver pathology. Further studies should provide greater knowledge for a better understanding of the protective mechanism(s) of VPA.

## Materials and methods

### Animals

The experiment was approved by the Research Ethics Committee, Biotechnology Research Center, Tripoli, Libya. Approval number (BEC-BTRC 8-2019). Methods were carried out in accordance with approved guidelines. All animal experiments were conducted in accordance with the ARRIVE guidelines and were performed per the National Institutes of Health Guiding Principles within the Care and Use of Animals. We used 30 adult Swiss male albino mice of 8 weeks old and weighing 27.5 ± 1.48 g. The mice were maintained under 12/12 h light/dark cycle at 26 ± 2 °C in the animal facility, Faculty of Sciences, University of Tripoli, Libya. All mice had free access to food and water.

### Experimental design

**Figure 6:**
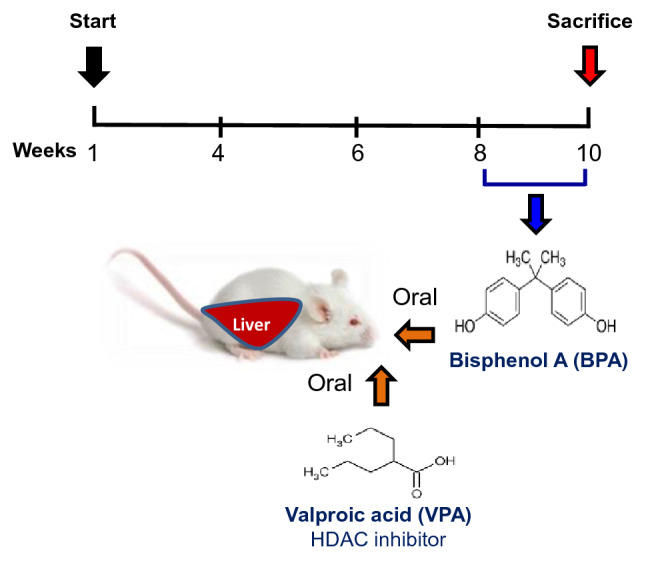
Schematic of animal treatment procedure. At 8 weeks old, mice were exposed to different treatments: sham, vehicle, VPA, BPA, or BPA + VPA. BPA and VPA were administered orally via gavage at a single dose of 4 mg/kg and 4 µg/kg for 2 weeks, respectively. At 10 weeks of age, the mice were euthanized and tissues were collected for biochemical and histological examinations. *VPA* valproic acid, *BPA* bisphenol A.

We randomly divided 30 mice into five groups: group 1: SC, normal control; group 2: VC, corn oil (vehicle control); group 3: BPA group, 4 mg/kg/day of BPA (single dose; Alfa Aesar, USA) in sterile corn oil; VPA group, 4 µg/kg bw VPA: VPA group (Sigma-Aldrich, Germany) twice a week for 2 weeks; and BPA + VPA group, BPA + VPA. BPA and VPA were administrated orally to mice via gavage. Once the mice were 10 weeks old, 24 h after the last treatment, the mice were euthanized for biochemical and histological examinations (Fig. 6). BPA doses were selected on the basis of earlier studies on the toxic effects of BPA^15,77–79^.

### Clinical assessment

Clinical observations were made every day. Any abnormal clinical signs or behaviors due to toxicity were recorded. Animals were monitored for their survival throughout the study. Overnight death of mice was recorded following morning and mortalities not related to exposure were determined by establishing cause of death by an independent observer.

### Body and liver weight measurement

The BWs were recorded before and after the end of the experimental duration, and initial and final BWs were used to calculate the BW change (%). Following the completion of treatment time livers were excised, weights were recorded and the relative BW (%) was determined.

### Blood samples

Blood samples from the inferior vena cava were collected in plain tubes for biochemical analysis. Serum was obtained by centrifugation at 3000 rpm for 15 min and stored at − 20 °C.

### Blood and tissue sample collection

The mice were euthanized via cervical dislocation under anesthesia with diethyl ether. Liver was quickly dissected out, washed and fixed in 10% buffered formalin for histological analysis. To determine lipid peroxidation and antioxidant values, pieces of the liver were frozen at − 20 °C until further use.

### Liver function indices in the sera

For liver function test, transaminases (alanine transaminase [ALT] and alkaline phosphatase [ALP]) were measured. The enzymatic-based method was used to measure both enzymes’ activities. ALP was assayed as previously described^80^ where phenylhydrazine formed is directly proportional to the enzyme quantity.

### Lipid peroxidation assay

Malondialdehyde (MDA) was quantified as a thiobarbituric acid (TBA)-reactive substrate, as described previously^81,82^. Briefly, liver tissue samples were homogenized in ice-cold 10% (w/v) phosphate-buffered saline using a tissue homogenizer (IKA, RW 20.n, Germany). The suspension was spun down, 500 µL aliquots of homogenate samples were added to 2 mL of trichloroacetic acid (TCA)-TBA, HCL reagent (15% TCA, 0.37% TBA, and 0.24 N HCl), and the mixture was boiled for 15 min and allowed to cool. Mixture was centrifuged and the obtained supernatant was read at 532 nm. To obtain a calibration curve, different amounts of 1,1,3,3-tetramethoxypropane were used as standard to measure concentration of TBA-MDA adducts and the result was expressed in nmol/mL.

### Protein peroxidation assay

The liver carbonyl protein content was measured spectrophotometrically using liver tissue, as described^83,84^. After the liver tissue samples were homogenized, where the final total protein content was adjusted to be less than 10 mg/mL, the samples were incubated for 1 h at room temperature in the dark with 10 mM 2-4-dinitrophenylhydrazine (DNPH) in 2.5 M HCl, in the same condition as the control samples. Protein was precipitated using 20% TCA, washed with ethanol–ethyl acetate (1:1), and redissolved using precipitated 6 M guanidine HCl. The DNP derivatization absorbance was measured at 370 nm using controls as a blank.

### Glutathione measurement

GSH levels were measured using Ellman’s reagent (5,5-dithiobis-(2-nitrobenzoic acid) [DTNB]), as described^85^. The liver homogenate was mixed with 25% TCA to precipitate proteins and centrifuged for 5 min at 4000 rpm. The supernatant was incubated with PBS (pH 7.4) and DTNB for 10 min. Absorbance was measured at 412 nm and the GSH levels were expressed as nmol/mg protein.

### Histopathological studies and microscopy

Liver tissue was prepared for histological preparation, as previously described^86,87^. Liver tissue samples were dehydrated in a series of upgrade concentrations of ethanol solutions, cleared in xylene, and embedded in paraffin wax. Next, 5-µm-thick paraffinized sections were cut, deparaffinized, hydrated, and stained with hematoxylin and eosin (H&E). Slides were examined under microscope (Leica Microsystems, Germany). The liver tissue sections from each mouse were assessed blind by a pathologist for histopathological changes.

Histopathologic investigation of liver tissue sections was performed with a focus on inflammatory changes, hepatocellular necrotic changes, biliary ductless changes, fibrosis, architecture changes, and regenerative changes. The frequency of lesions was unbiased evaluated to determine a pattern of injury and assessed semi-quantitatively according to the severity and distribution of the pathological changes.. Only diagnoses that were considered accurate based on histopathological changes and the data presented in each group were included in the histopathological description and depictions. The degree of severity of lesions was assessed as minimal (1 focus or less with 10 × objective), mild (2–4 foci with 10 × objective), moderate (5–10 foci with 10 × objective), or severe (> 10 foci with 10 × objective) according to the modified Ishak score^88^. Grading calculation of these assigned numbers to assess the severity of the necro-inflammatory features was performed, as previously described^88^. Locations without lesions were designated as normal (none). The histopathological findings were divided according to lesion possibility, staining, and histological components of the liver with reference to the detailed algorithm of Batts and Ludwig^89^. The four criteria of scoring were averaged and considered as a replicate.

### Cell death scoring

Cell death was assessed using image-analysis ImageJ software (National Institutes of Health, MA, USA; http://rsb.info.nih.gov/ij/). Cell numbers from H&E stained liver tissue slides were calculated as a percentage of number of cells present and overall average of the cell numbers obtained from each treatment group).

Apoptotic cells were characterized by their morphology of cell shrinkage; in addition, other cell properties were assessed, such as chromatin condensation, cellular migration rate, and apoptotic body identification^54^. Hepatocyte necrosis was assessed using the following criteria: increased cytoplasmic eosinophilia, cell swelling, karyorrhexis, and karyolysis. The apoptosis percentage was determined by counting the number of apoptotic hepatocytes throughout 40 × objective microscopic fields out of the entire histologic section. The scoring scale, 0 to 5 was set based on no necrosis, minimal, mild to moderate, moderate, moderate to severe, and severe, respectively. For quality of scoring, the final score of each liver tissue section was calculated as an average of eight scores.

### Statistical analysis

The Data were analyzed using GraphPad Prism software version 7.0. All data represent the mean ± SEM. Also, normality was assessed using the computerized Kolmogorov–Smirnov test. For data with normal distributions, single intragroup comparisons were made using Student’s paired *t* test and multiple comparisons were made using one-way analysis of variance, followed by a post hoc test for multiple comparisons. Dunnett’s multiple-comparison test was used to detect pairwise intergroup differences. P < 0.05 was set as statistically significant.

## Data Availability

Data used during the current study are available from the corresponding author.
